# Cardiac Arrest After Adenosine Administration in Compensatory Tachycardia: A Case Report

**DOI:** 10.7759/cureus.54780

**Published:** 2024-02-23

**Authors:** Craig R Torres-Ness, Sonia A Desai

**Affiliations:** 1 Emergency Medicine, University of Southern California Keck School of Medicine, Los Angeles, USA; 2 Emergency Medicine, Los Angeles County University of Southern California Medical Center, Los Angeles, USA

**Keywords:** paroxysmal supraventricular tachycardia, emergency medical services, compensatory tachycardia, prehospital, cardiac arrest, adenosine, case report

## Abstract

Compensatory tachycardia, an increased heart rate responding to stressors, requires careful consideration in treatment. This case report outlines a scenario where emergency medical services (EMS) misinterpreted a patient’s electrocardiogram (EKG) as paroxysmal supraventricular tachycardia (PSVT) and administered adenosine, resulting in sudden cardiac arrest. Despite the rarity of deaths post-adenosine, this case highlights the potential risks of its use in inappropriate clinical scenarios. The patient, later diagnosed with a pulmonary embolism, had a compensatory heart rate that was disrupted after adenosine administration. While adenosine remains a safe and effective treatment for PSVT, this case report serves as a warning to EMS systems about the risks associated with its increased misuse, especially given the trends of prehospital EKG misinterpretation.

## Introduction

Compensatory tachycardia is defined as an increased heart rate in response to an underlying stressor, such as an acute illness, where the sympathetic tone is increased. In these patients, an increased heart rate is often necessary to maintain adequate cardiac output for hemodynamic stability [1]. Typically, the underlying rhythm is normal sinus rhythm, but not always. For example, in patients with atrial fibrillation, a rapid ventricular rate may be in response to a stressor such as dehydration or infection. This is in contrast to paroxysmal supraventricular tachycardia (PSVT), a reentrant conduction abnormality resulting in increased heart rate [2]. In patients presenting with compensatory tachycardia, treatments must focus on addressing the underlying cause, as simply slowing the heart rate can be detrimental. In PSVT, maneuvers and treatments are focused on rate control and attempt to disrupt and reset the circuit abnormality. The distinction between these types of tachycardic rhythms is well known to clinicians, but patient presentations are often complex, dynamic, and unpredictable.

In this case report, we present a clinical scenario where a patient was in respiratory distress and had an electrocardiogram (EKG) misinterpreted as PSVT by emergency medical services (EMS). Given this, the prehospital personnel utilized an Advanced Cardiovascular Life Support (ACLS) protocol focused on PSVT by slowing down the rate using both vagal maneuvers and intravenous (IV) adenosine. Unfortunately, the patient’s tachycardia was in response to an underlying, acute illness and was necessary for her to maintain adequate hemodynamics. Immediately following the administration of adenosine, the patient went into cardiac arrest. In our review of the literature, there have been only three documented cases of death following adenosine administration in the prehospital setting and none in the emergency department [3,4]. Given the prevalence of use by EMS, this case serves as an important reminder of the potential consequences of adenosine administration in the incorrect clinical scenario and stresses the importance of accurate prehospital EKG interpretation and correct patient selection to prevent adverse outcomes.

## Case presentation

**Figure 1:**
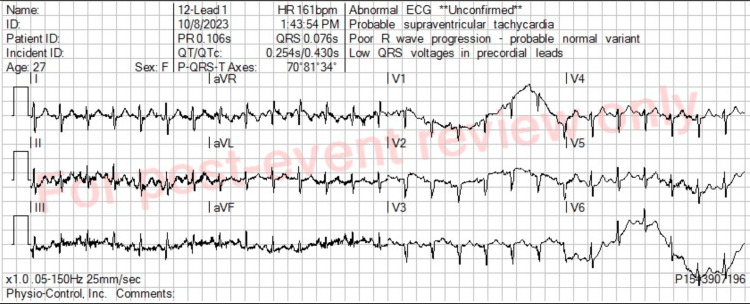
Prehospital EKG with software interpretation

A 27-year-old female with no known past medical history reportedly had worsening palpitations and shortness of breath for one week. Per family, the patient had been to two urgent care centers over the past week, but no diagnosis or effective treatment was provided. At the time of the 911 call, the patient had awakened from a nap feeling markedly short of breath, weak, and anxious. On EMS assessment, she was found to be in severe distress with labored breathing and was noted to be pale and diaphoretic. She had a blood pressure of 125/99, and her remaining initial vital signs were significant for a heart rate of 165, respiratory rate of 26, and oxygen saturation of 79% on room air. The patient was placed on a non-rebreather mask (NRB) at a flow rate of 15 L/min, with improvement in her oxygenation to a saturation level of 90%. An 18-gauge IV was placed in the patient’s left antecubital fossa. Following this, a 12-lead EKG was obtained, and the EKG software interpretation read “probable supraventricular tachycardia” (Figure 1). Paramedic personnel also interpreted the EKG as PSVT. Per ACLS protocol, a Valsalva maneuver was attempted on the scene, and the patient’s heart rate briefly lowered to 59 for a few seconds but subsequently returned to a rate of 159. Also noted during the brief bradycardic episode was a drop in her oxygenation to a saturation of 79%, which then improved to 88%. Given the ineffectiveness of the Valsalva maneuver, the paramedic personnel contacted the designated hospital base station for further guidance. The paramedic expressed concern for an unstable PSVT, given the patient’s respiratory distress. The base station’s Mobile Intensive Care Nurse (MICN), who is specially trained to provide prehospital clinical support, followed treatment guidelines for PSVT with poor perfusion and recommended a 6 mg IV bolus of adenosine. It is important to note that the EKG was not electronically transmitted, and the MICN was acting on verbally reported PSVT. In addition, our system’s prehospital protocol does not require authorization for adenosine, but the MICN was instead contacted for decision support. The adenosine was given by the paramedics as the ambulance was arriving at our emergency department. Immediately after administration, the patient became unconscious and apneic with no palpable pulse or blood pressure.

Cardiopulmonary resuscitation (CPR) was initiated, and the patient arrived at the emergency department with an initial rhythm of pulse electrical activity (PEA) and was immediately intubated. A secondary physical exam, point-of-care ultrasound (POCUS), and point-of-care chemistry panel were performed with no significant reversible etiologies or severe electrolyte derangements identified. Given the clinical presentation of this young patient’s witnessed cardiac arrest, three differential diagnoses were considered as most likely: hypoxic respiratory failure, pulmonary embolism, and medication-induced tachyarrhythmia. 

The striking temporal relationship between adenosine administration and cardiac arrest prompted consideration of the possibility of Wolff-Parkinson-White (WPW) syndrome but was quickly ruled out as the EMS EKG did not exhibit any concerning WPW features. Instead, two emergency medicine physicians confirmed the rhythm as sinus tachycardia with a rate of 161 beats/min, rather than previously interpreted PSVT. The resuscitation continued, and given the concern for pulmonary embolism, the patient was given a 50 mg IV bolus of tissue plasminogen activator (TPA), followed by another 50 mg IV bolus of TPA 10 minutes later. Unfortunately, despite prolonged resuscitative efforts, a return of spontaneous circulation was not obtained, and the patient was pronounced dead. An autopsy report later revealed the cause of death to be pulmonary embolism with associated deep-leg vein thrombosis.

## Discussion

This case underscores the potential risks associated with incorrect adenosine administration in patients with compensatory tachycardia due to underlying critical illness. Mirroring a prehospital case report published over two decades ago by Haynes, our patient with presumed right heart strain and tenuous hemodynamics secondary to a large pulmonary embolism also suffered cardiac arrest immediately after prehospital adenosine administration [3]. The brief pause in cardiac output from the adenosine caused an interruption of her compensatory hemodynamic response, which very likely contributed to her subsequent cardiac arrest [3]. A significant strength of our case report is the robust documentation and reporting provided by the prehospital personnel. These thorough records, along with the EKG captured in the field, delineate a clear clinical progression. This, coupled with the autopsy report, provides an understanding of what transpired. While this certainly does not confirm that adenosine was the cause of death, the contemporaneous relationship does support that it likely hastened the cardiovascular collapse. The goal of this report is to gain an understanding and appreciation of the risk versus benefit ratio of adenosine administration in prehospital settings is.

The American Heart Association (AHA) and the American College of Cardiology (ACC) professional guidelines recommend IV bolus administration of adenosine as first-line pharmacotherapy for hemodynamically stable PSVT that does not respond to vagal maneuvers [5]. Its effectiveness at terminating PSVT within seconds and returning the patient to normal sinus rhythm is well established. In addition, the medication is considered extremely safe. A major contributor to the favorable safety profile of adenosine is the relatively short half-life of less than 10 seconds. Minor side effects include flushing, dyspnea, chest pain, feeling of doom, and headache. However, more severe side effects, such as bronchospasms, hypotension, and dysrhythmias, are well documented in the literature [6,7]. While the side effects of adenosine are well-documented, their brevity is usually considered protective.

Given adenosine’s favorable drug profile, studies from the early 1990s helped to establish this as a treatment option in the prehospital setting. Specifically, these studies showed that paramedics could adequately identify PSVT and that the administration of adenosine in the prehospital setting was safe for PSVT rhythm termination [8,9]. Subsequent research investigated protocols that removed the physician from being involved in the management of these types of patients, and prehospital treatment algorithms across the United States integrated adenosine as a standing order, as opposed to requiring a physician’s authorized approval to administer [10]. As the use of adenosine has increased substantially in the prehospital setting, adverse patient outcomes are exceedingly uncommon and rarely reported [11,12]. 

All that said, a trend since the implementation of adenosine as a standing order in the prehospital setting is the increased frequency in which rhythms are misidentified and adenosine administered to patients where it was not clinically indicated [12]. From the original studies in the early 1990s, using single-lead EKGs, paramedics were said to accurately identify PSVT 79.2% of the time. Subsequent studies have paramedic misdiagnosis rates ranging between 21% and 43%, and a recently published study from a large urban EMS system had a paramedic PSVT misdiagnosis rate between 45% and 50% [9,10,13,14]. Despite the frequency in which rhythms were misinterpreted in these studies, including the times in which adenosine was administered to patients without PSVT, there were no sustained adverse patient outcomes. This may be why the misidentification rates remain so high. Although the repercussions may not be immediately apparent to prehospital personnel and emergency physicians, the misidentification rate does lead to delays in definitive diagnosis, life-threatening consequences, and, as outlined in our case report, cardiac arrest.

Following the publication of Haynes’ case reports on cardiac death after adenosine, there have been no other published reports of death following the administration of adenosine in the prehospital setting [3]. Perhaps the warnings were heeded, patient selection refined, rhythm interpretation improved, and there truly have been no adverse patient outcomes related to this prehospital administration. However, given the propensity for it to be given in patients with tachycardia that is not a reentrant conduction abnormality, there is a strong possibility that it is given to patients whose hemodynamics require compensatory tachycardia. If this is the case, it also means that the clinical assessment of the patient is potentially incorrect, and therefore, other appropriate treatments are delayed. While these adverse effects of this delay are difficult to capture and measure, it can be easily deduced that if a patient with sinus tachycardia is given adenosine, the underlying cause of the sinus tachycardia has not been identified.

Sternbach wrote an editorial in 2001 in response to two patient reports of death following adenosine and stated, “The availability of a rapid-acting agent intended to correct one rhythm may readily lead to its improper use for others” [15]. In addition, he went on to state that despite its significant safety profile, this should not be misinterpreted as it can be administered without the risk of harm [15]. Most of the time, adenosine use is appropriate and without much harm, apart from the transient side effects. However, this case report highlights that adenosine, like all medications, should not be given without full consideration of the potential side effects. And that the lack of published adverse outcomes should not falsely reassure providers into complacency. Instead, clinical assessment should remain the main driver in deciding what, if any, intervention is needed. And since so much of PSVT treatment rests on the interpretation of the EKG, prehospital settings should pay close attention to the trend toward misinterpreting EKGs as an impetus to enhance training protocols.

## Conclusions

Adenosine is a safe and effective treatment for PSVT, and this case report should not change providers’ approaches to treating it. However, given the increased use of adenosine in the prehospital setting, coupled with trends indicating that EKG misinterpretation is increasing, this case should serve as a warning to EMS systems that adenosine administration is not without risk. Furthermore, in patients whose tachycardia is compensatory, clinical assessments should focus on the underlying etiology of the increased heart rate, as adenosine is not indicated in these cases. While this case report should not change established, evidence-based practices, our hope is that it serves as a caveat that no medication administration is without risks, and judicious use is imperative.
